# Supplementary figures and images for: Optimizing interneuron circuits for compartment-specific feedback inhibition
Source: PLoS Comput Biol. 2022 Apr 28;18(4):e1009933. doi: 10.1371/journal.pcbi.1009933 (PMC9049365; doi:10.1371/journal.pcbi.1009933)

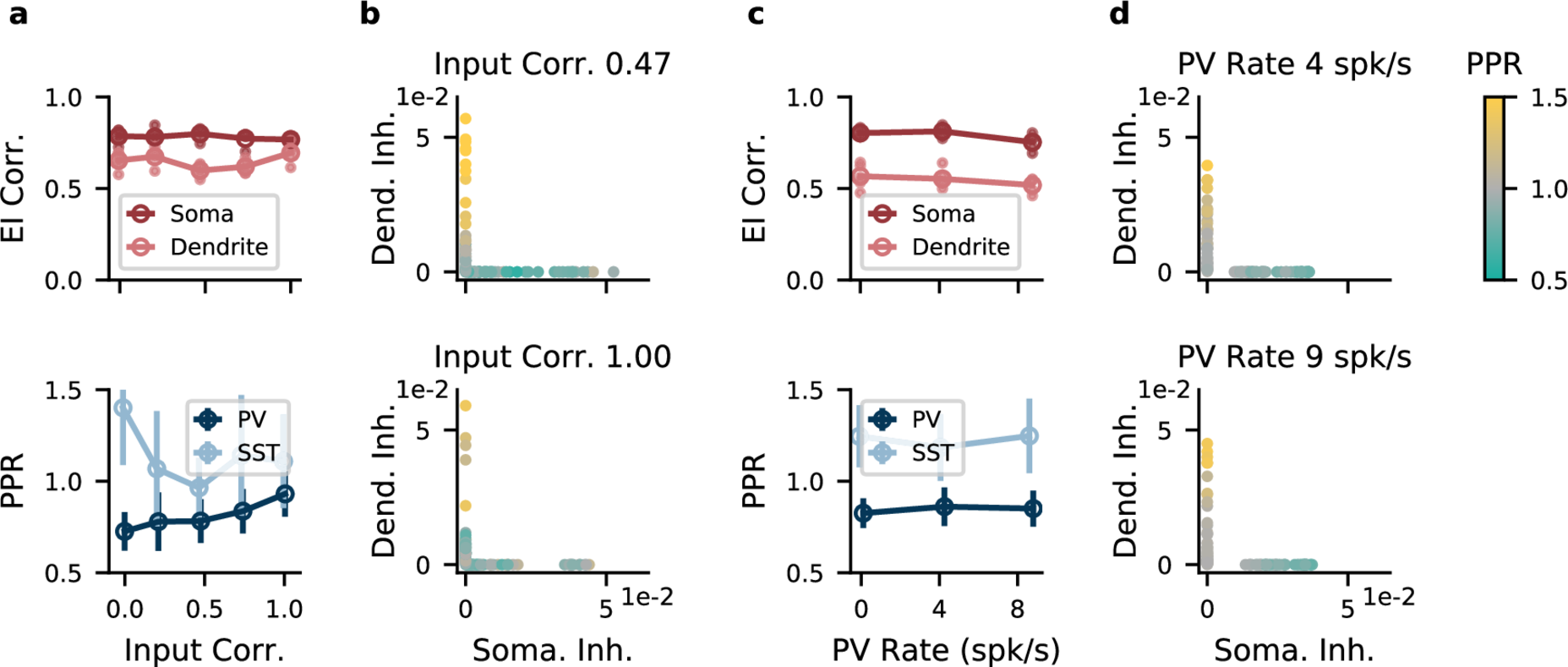

Supplement: S1 Fig — A: Top, performance as measured by compartment-specific correlation between excitation and inhibition of networks trained on different correlations between compartment-specific excitatory inputs. Open circles, mean over 5 batches of 8 stimuli with random amplitudes (see Methods). Small filled circles, individual batches. Here and in the other panels, the interneurons were assigned to inhibit only the soma or only the dendrites. Bottom, interneuron specialization as measured by Paired Pulse Ratio (PPR) decreases with input correlations. Error bars denote sd over IN populations. B: Strength of somatic and dendritic inhibition from individual INs. Top, medium input correlation (0.47); bottom, high input correlation (1.00). Color indicates PPR. C: Top, as A but as function of minimum PV rate. Bottom, interneuron specialization as measured by Paired Pulse Ratio (PPR) is not influenced by minimum PV rate. D: Strength of somatic and dendritic inhibition from individual INs. Top, medium PV rate (4 spk/s); bottom, high PV rate (9 spk/s). (TIF) [file pcbi.1009933.s001.tif]

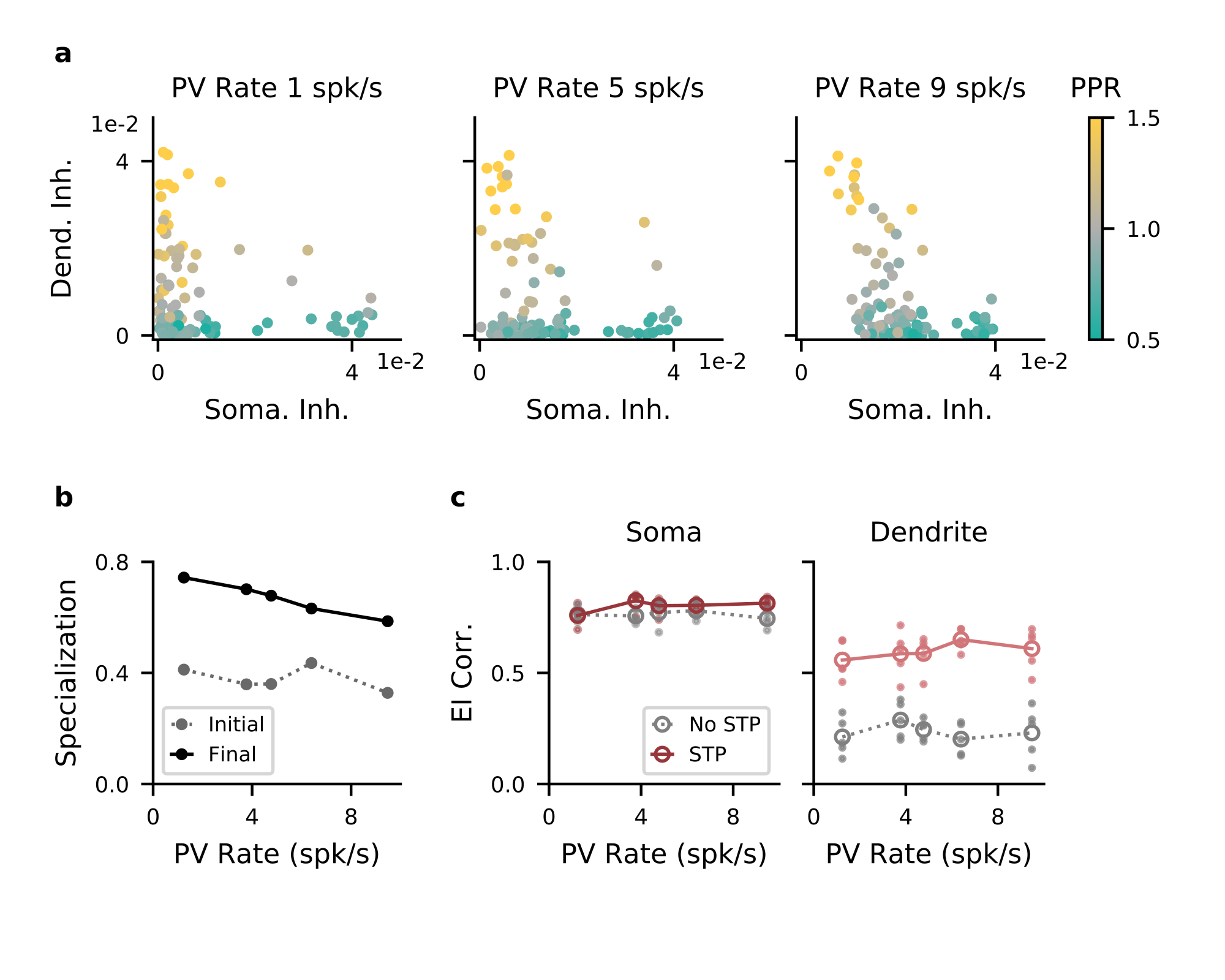

Supplement: S2 Fig — A: Strength of somatic and dendritic inhibition from individual INs. Left, middle, right: network optimized with a baseline PV rate of 1 (low), 5 (medium), and 9 spk/s (high), respectively. B: Specialization of IN→E weights. If each IN targets either soma or dendrites, the specialization is 1 (see Methods). Gray: specialization of initial, random network; black: specialization after optimization. C: Left, correlation between excitation and inhibition as function of minimum PV rate. Red: networks with optimized short-term plasticity. Gray: Networks without short-term plasticity. Open circles, mean over 5 batches of 8 stimuli with random amplitudes. Small filled circles, individual batches. (TIF) [file pcbi.1009933.s002.tif]

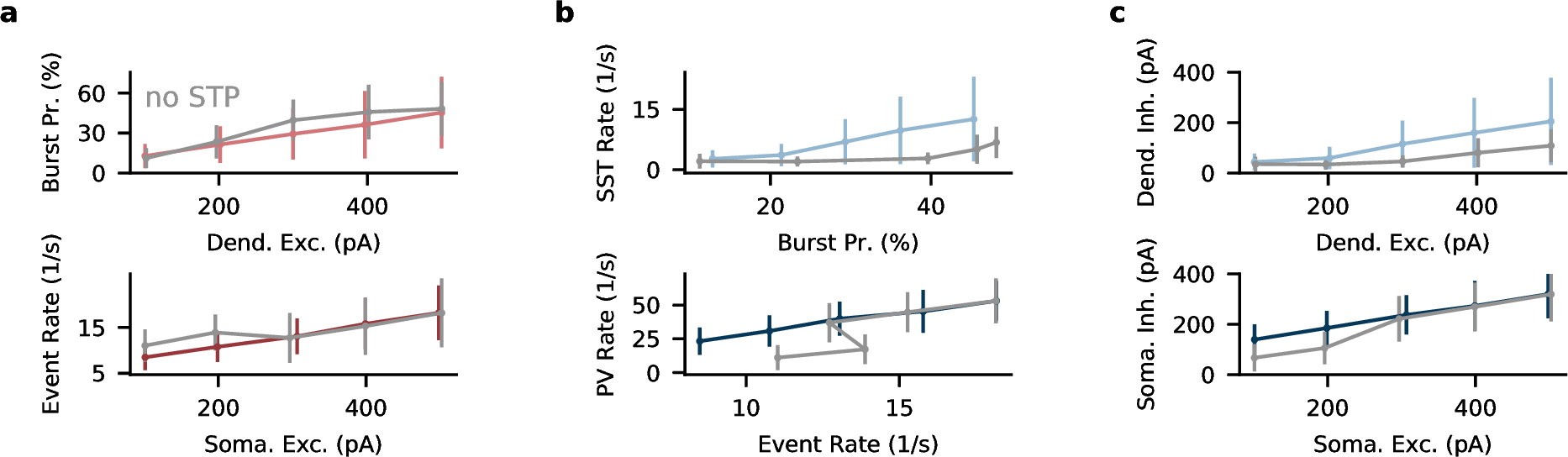

Supplement: S3 Fig — A: PCs use a multiplexed neural code both in presence (colors) and absence (gray) of short-term plasticity in their efferents. Top: Excitatory input to PC dendrites increases burst probability. Bottom: Excitatory input to PC somata increases event rate. B, top: SST rate increases with bursts probability only when SSTs receive short-term plastic input. Bottom: PV rate increases with PC events, but absent short-term plasticity only for intermediate and high event rates. C, top: dendrite-specific inhibition requires short-term plasticity. Bottom: soma-specific inhibition requires short-term plasticity only for small somatic input. (TIF) [file pcbi.1009933.s003.tif]

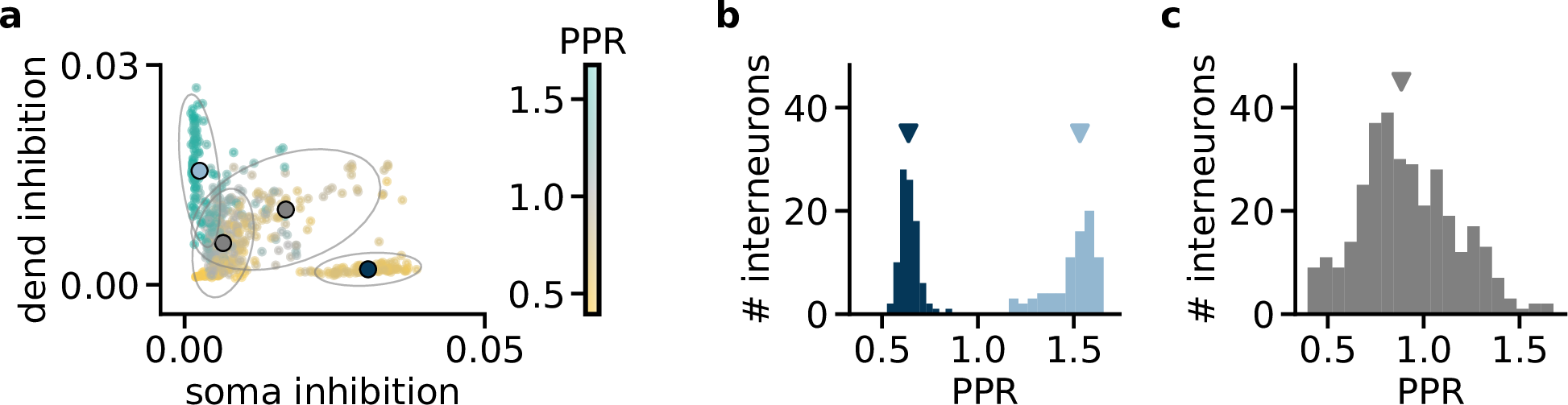

Supplement: S4 Fig — A: IN→PC weights after optimization, in networks where INs can connect to each PC soma (and dendrite) with a unique strength. Shown are the per-IN weights averaged over all PCs. A Gaussian mixture model identified 4 clusters: a PV and a SST cluster and 2 unspecific clusters. Dots show means, ellipses show 95% density. The PV and SST clusters contain 19% and 15% of the interneurons, respectively. The unspecific clusters with small and large weights contain 52% and 14% of interneurons, respectively. B: PV and SST interneurons receive depressing and facilitating inputs, respectively, as measured by the average paired pulse ratio (PPR), computed over all presynaptic PCs. Arrows indicate means. C: As B, but for interneurons of the two unspecific clusters. Unspecific interneurons do not receive a particular type of short-term plastic input. (TIF) [file pcbi.1009933.s004.tif]
