# Supplementary material for: Optimizing interneuron circuits for compartment-specific feedback inhibition
Source: PLoS Comput Biol. 2022 Apr 28;18(4):e1009933. doi: 10.1371/journal.pcbi.1009933 (PMC9049365; doi:10.1371/journal.pcbi.1009933)
Supplement: S1 Table — (PDF) [file pcbi.1009933.s005.pdf]

S1 Table. Network parameters

Symbol	Value	Unit	Description
N_E	400	-	Number of exc. neurons
N_I	100	-	Number of inh. neurons
E_L	-70	mV	reversal and reset potential
ϑ	-50	mV	spiking threshold
$\tau_{s/d/i}$	16 / 7 / 10	ms	time const. soma/ dend./inh. membrane
τ_r	3	ms	refractory time soma and inh.
$g_{s/d}$	1300 / 1200	pA	Coupling from dend to soma
$C_{s/d/i}$	370 /170/100	pF	Conductance of soma/dend./inh.
$\tau_{s/d,w}$	100 / 30	ms	Time const. adaptation soma/dend.
b_s	-200	pA	Spike-triggered adaptation (soma)
a_d	-13	nS	Voltage-driven adaptation (dend)
c_d	2600	pA	Coupling soma to dend.
E_d	-38	mV	position dend. nonlinearity
D_d	6	mV	steepness of dend. nonlinearity
$\mu_{s/d/i}$	400 / -300 /-100	pA	mean background input soma/dend./inh.
$\sigma_{s/d/i}$	450 / 450 / 400	pA	sd background input
τ_{bg}	2	ms	time const. background input
τ_{syn}	5	ms	time const. synapses
τ_u	100	ms	time const. facilitation
τ_R	100	ms	time const. depression
F	0.1	-	facilitation jump
