# Supplementary material for: Optimizing interneuron circuits for compartment-specific feedback inhibition
Source: PLoS Comput Biol. 2022 Apr 28;18(4):e1009933. doi: 10.1371/journal.pcbi.1009933 (PMC9049365; doi:10.1371/journal.pcbi.1009933)
Supplement: S2 Table — (PDF) [file pcbi.1009933.s006.pdf]

S2 Table. Optimization parameters

Symbol	Value / Init. Distribution	Dimensions	Description
U	$\mathcal{U}(0.1, .25)$	$N_E \times N_I$	Initial release prob.
$W^{E\to I}$	$\mathcal{N}(0, 1/N_E)$	$N_E \times N_I$	Exc. to Inh. weight
$W^{I\to I}$	$\mathcal{N}(0, 1/N_I)$	$N_I \times N_I$	Inh. to Inh. weight
$W^{I\to D}$	$\mathcal{N}(0, 0.2/N_I)$	$N_I \times 1$	Inh. to Exc. Dend. weight
$W^{I\to S}$	$\mathcal{N}(0, 0.2/N_I)$	$N_I \times 1$	Inh. to Exc. Soma weight
-	1e-3	-	learning rate for weights
-	4e-3	-	learning rate for U
β	10	-	Slope spiking derivative
-	1.0	-	Gradient (absolute value) clipping
