# Supplementary material for: Optimizing interneuron circuits for compartment-specific feedback inhibition
Source: PLoS Comput Biol. 2022 Apr 28;18(4):e1009933. doi: 10.1371/journal.pcbi.1009933 (PMC9049365; doi:10.1371/journal.pcbi.1009933)
Supplement: S1 Appendix — (PDF) [file pcbi.1009933.s007.pdf]

S1 Appendix. Mathematical Analysis of a Simplified Network Model
We performed a mathematical analysis of a simplified network to better understand the following results of our spiking network simulations:

1. A compartment-specific balance requires PV $\rightarrow$ SST inhibition, but no other IN $\rightarrow$ IN connectivity (Fig 5).

2. Higher interneuron rates require less IN specialization, i.e., individual interneurons often inhibit both PC compartments (Fig 4).

The simplified model consists of a population of principal cells (PC) and two populations of interneurons that we will refer to as parvalbumin (PV)-positive and as somatostatin (SST)-positive cells. The population activity of the PCs is represented by somatic activity e and dendritic activity b. The interneuron activities are represented by firing rates p and s. The four activity variables e, b, p, s are best thought of as deviations of the respective activity from baseline. The activity variables can hence be both positive and negative (ignoring saturation effects that arise when the baseline is very low, see below).

For our analysis, we make the following assumptions: (1) somatic input linearly increases somatic activity e, (2) dendritic input linearly increases dendritic activity b, which is in turn assumed to be independent of somatic input/activity (note that the latter assumption deviates from a BAC-firing mechanism [31], but is necessary to obtain a linear model), (3) the activities p, s of the interneuron populations increase linearly with their input, and (4) short-term plasticity is characterized by a single, static parameter (see below). Because we are interested only in qualitative statements, the analysis is done in terms of unitless variables. The model describes the dynamics of the four activity variables e, b, p, s:

$$\dot{e} = -e - w^{ep}p + E^e(t), \tag{19}$$

$$\dot{b} = -b - w^{bs}s + E^b(t), \tag{20}$$

$$\dot{p} = -p + \alpha w^{pe}e + (1-\alpha)w^{pe}b - w^{ps}s, \tag{21}$$

$$\dot{s} = -s + \beta w^{se}e + (1-\beta)w^{se}b - w^{sp}p. \tag{22}$$

Here, the synaptic weight from population y to x is modeled with a non-negative weight w^{xy} ($x, y \in \{e, p, s\}$; e: PCs, p: PV INs, s: SST INs). The central tenet of this simplified model is that somatic and dendritic activity both generate characteristic spike patterns in PCs—such as events and bursts—which are selectively transmitted by synapses because of short-term plasticity. The parameters $\alpha, \beta \in [0, 1]$ describe the short-term plasticity of the PC$\rightarrow$PV and PC$\rightarrow$SST synapses, respectively. $\alpha, \beta = 1$ corresponds to synapses that only transmit somatic activity. If somatic activity generates events and dendritic activity generates bursts, this would require "perfectly depressing" synapses, i.e., synapses that transmit only the first spike of a burst. $\alpha, \beta = 0$ corresponds to synapses that only transmit dendritic activity. For the case where dendritic activity generates bursts, this requires "perfectly facilitating" synapses that ignore individual spikes and transmit only bursts. We assumed that the projections of the interneurons are specialized, i.e., that PV interneurons inhibit the soma and SST interneurons inhibit the dendrite. We will abandon this assumption in Section Influence of IN baseline firing rates on interneuron specialization. We also excluded inhibitory recurrence within the two populations (PV $\rightarrow$ PV, SST $\rightarrow$ SST), because these connections would only change the effective time constant of the respective activation variable. The somata and dendrites of the PCs receive time-varying external inputs $E^e(t)$ and $E^b(t)$, respectively. All activity variables follow leaky dynamics.

The dynamical system can be written as $\dot{r} = Wr + I$, where the vector r contains the activation variables $r = (e, b, p, s)^T$, I contains the external inputs

$I = (E^e, E^b, 0, 0)^T$, and W is the matrix of effective connectivity strengths

$$W = \begin{pmatrix} -1 & 0 & -w^{ep} & 0 \\ 0 & -1 & 0 & -w^{bs} \\ \alpha w^{pe} & (1-\alpha)w^{pe} & -1 & -w^{ps} \\ \beta w^{se} & (1-\beta)w^{se} & -w^{sp} & -1 \end{pmatrix}. \tag{23}$$

Assuming that the time constant of the network is sufficiently short to adiabatically follow the input currents, we can consider the steady state by setting $\dot{r} = 0$ and solving for r:

$$Wr + I = 0 \implies r = -W^{-1}I. \tag{24}$$

Influence of IN→IN connections on compartment-specific E/I balance

In the steady state Eq. (24), the IN rates are equal to

$$p = -[W^{-1}]_{31}E^e - [W^{-1}]_{32}E^b, \tag{25}$$

$$s = -[W^{-1}]_{41}E^e - [W^{-1}]_{42}E^b. \tag{26}$$

Here, $[W^{-1}]_{ij}$ refers to the element in row i and column j of the matrix W^{-1}. Assuming that the interneurons specialize by inhibiting a single compartment, a necessary (and, up to scaling, sufficient) condition for compartment-specific balance is that the PV rate p is proportional to the external input targeting the soma and independent of the input targeting the dendrite. Similarly, the SST rate should be proportional to the external input targeting the dendrite and independent of the input targeting the soma. By Eq (25), a compartment-specific balance hence requires $[W^{-1}]_{32} = 0$ and $[W^{-1}]_{41} = 0$. Computing these matrix entries yields:

$$[W^{-1}]_{32} \propto w^{pe}(1-\alpha) - w^{ps}w^{se}(1-\beta)) = 0 \tag{27}$$

$$[W^{-1}]_{41} \propto -w^{pe}w^{sp}\alpha + w^{se}\beta = 0\,. \tag{28}$$

These equations have a simple interpretation. Each of the two terms in $[W^{-1}]_{32}$ represents a pathway by which dendritic activity reaches the PV interneurons. The first term quantifies how much dendritic activity reaches PV interneurons via the direct excitatory PC→PV projection, the second represents corresponding feedforward inhibition via the PC→SST→PV pathway. If these two pathways cancel, PV activity is independent of dendritic activity. Similarly, the two pathways in $[W^{-1}]_{41}$ that transmit somatic activity to the SST need to cancel.

What is the role of short-term plasticity? For illustration, let us first consider the limiting case of "perfect" synaptic depression ($\alpha = 1$). Perfectly depressing PC → PV synapses would imply that the PV interneurons only receive somatic activity from the PCs via the direct PC→PV pathway. The condition (27) then reduces to

$$[W^{-1}]_{32} \propto -w^{ps}w^{se}(1-\beta) = 0\,, \tag{29}$$

i.e., dendritic activity should not reach PV interneurons via the indirect PC→SST→PV pathway, because this would render PV activity dependent on dendritic activity. Because dendritic activity need to be transmitted to the SST interneurons to reach an E/I balance in the dendrite, this implies that the SST→PV connection should be absent.

"Perfect" synaptic depression ($\alpha, \beta = 1$) or facilitation ($\alpha, \beta = 0$) are hard to implement, certainly by a Markram-Tsodyks model in the presence of background activity. However, the effect of imperfect depression in the PC→PV connection ($\alpha < 1$)

can be compensated by feedforward inhibition along the PC→SST→PV pathway. Similarly, imperfect PC→SST facilitation picks up somatic activity, which can then be canceled by feedforward inhibition via the PC→PV→SST pathway. The role of IN→IN synapses is therefore to complement "imperfect" short-term plasticity in decoding compartment-specific inputs.

The observation that PV→SST connections are the most important IN→IN connections in our model results from "imperfect" facilitation in the excitatory synapses onto SST interneurons. Because events occur more frequently than bursts, the excess excitation they trigger in SST interneurons needs to be actively cancelled via the PV→SST pathway. The converse SST→PV connection is less critical, because bursts are comparatively rare, such that their transmission via PC→PV synapses causes only minor disturbances of the compartment-specific E/I balance.

Influence of IN baseline firing rates on interneuron specialization

The previous analysis assumed that interneurons were specialized to inhibit a single compartment. When should we expect specialization in the first place? We can investigate this question by extending the simplified model by inhibition from all INs onto all PC compartments:

$$W = \begin{pmatrix} -1 & 0 & -w^{ep} & -w^{es} \\ 0 & -1 & -w^{bp} & -w^{bs} \\ \alpha w^{pe} & (1-\alpha)w^{pe} & -1 & -w^{ps} \\ \beta w^{se} & (1-\beta)w^{se} & -w^{sp} & -1 \end{pmatrix}. \tag{30}$$

A compartment-specific balance now requires external input to be canceled by the inhibition from both interneurons:

$$E^e = w^{ep}p + w^{es}s, \tag{31}$$
$$E^b = w^{dp}p + w^{ds}s. \tag{32}$$

Without additional constraints, this system has an infinite number of solutions, i.e., weight configurations that achieve a compartment-specific balance. However, the simple constraint of low baseline firing rates of the interneurons collapses the solution space to the specialized one ($w^{es} = w^{dp} = 0$), for the following reason.

The activity variables p, s represent deviations of the interneurons firing rates from baseline. If the baseline is sufficiently high, these deviations can be both positive and negative. In that case, inhibition from one interneuron class can be cancelled by disinhibition from the other interneuron class. PV and SST interneurons are then both free to respond to both somatic and dendritic activity, as long as the weighted sums of the inhibition and disinhibition they provide to PC somata and dendrites mirrors the excitatory input to those compartments. There are many ways of doing so.

For low baseline firing rates, disinhibition is no longer available, because negative deviations from baseline are limited by the fact that activities cannot be negative. For illustration, let us consider the case where both PV and SST neurons have zero baseline activity. By definition, the excitatory signals $E^{e/b}$ are zero for baseline activity, because they represent deviations from baseline. Therefore, we can assume that there exists a moment where the input to one PC compartment is zero, while the input to the other compartment is positive (e.g., $E^e = 0$, $E^b > 0$). By Eq. (31) and because all weights and rates must be positive, $w^{ep}p + w^{es}s = 0$ implies that $w^{ep} = 0$ or $p = 0$ and $w^{es} = 0$ or $s = 0$. At least one weight has to be non-zero (otherwise balancing the soma is impossible), and at least one rate has to be non-zero (otherwise balancing the dendrite is impossible). Without loss of generality we can conclude that $w^{ep} > 0, p = 0, w^{es} = 0,$

and $s > 0$: The PC soma is only inhibited by the PV neuron. Analogously, the existence of a moment when $E^b = 0$ but $E^e > 0$ implies that $w^{ds} > 0, w^{dp} = 0$, meaning that the PC dendrite is only inhibited by the SST neuron. If the baseline activity is low, but not strictly zero, this saturation arguments still hold, if the variations in the firing rates that are required to balance the external input are larger than the baseline activity. Low baseline firing rates therefore imply interneuron specialization, because they prevent inhibition and disinhibition from non-specialized neurons to cancel.
