# Supplementary material for: Optimizing interneuron circuits for compartment-specific feedback inhibition
Source: PLoS Comput Biol. 2022 Apr 28;18(4):e1009933. doi: 10.1371/journal.pcbi.1009933 (PMC9049365; doi:10.1371/journal.pcbi.1009933)
Supplement: Attachment — Submitted filename: keijser-sprekeler.pdf [file pcbi.1009933.s008.pdf]

The authors proposed a normative explanation for the diversity of inhibitory interneurons in the cortex. More precisely, they hypothesize that different interneurons types emerge from the objective of balancing excitation and inhibition at a compartmental level. The authors test the feasibility of their hypothesis using an optimization approach, and show that in some cases two clusters (one per model neuron compartment) emerge from an initially homogeneous group of neurons. The main result is illustrated in Fig. 1 and most of the paper is dedicated to understanding the underlying mechanisms and to the robustness of this phenomenon.

One of the most interesting observations in my opinion is that to achieve the objective, interneurons should be able to "invert" the spiking signal to obtain the membrane potential. This problem is elegantly solved in the model by specializing different interneurons to read out only bursts or only spikes thanks to short-term plasticity. However, it should be pointed out that this idea is not entirely new, as the authors themselves reference previous work from the same group (Naud and Sprekeler, 2018), in which the same basic mechanism was already described. *The main new contribution of this paper is then the application of this mechanism to the compartment specific balance of excitation and inhibition.* I see a few major problems with the conclusions that the authors are drawing:

- The authors stress that the network was not optimized to read out these two signals, and that this emerged automatically from the goal. However, if I understood correctly, because of the way the optimization was set up, reading out burst and spikes differentially is the only way the problem can actually be solved, so the two goals are actually equivalent. It would be interesting, or at least useful for clarification, to test the following:
  - Are there any other solutions of the compartment specific E-I balance that do not involve short-term plasticity? This could be easily tested by removing STP from the optimization
  - Would two clusters of interneuron emerge in a heterogeneous network in which the inhibitory connectivity is allowed to take any value (and not the same for all neurons)?
- In the discussion, the authors ask whether it would be possible to extend their results to multiple interneuron classes by exploiting more complex neuronal morphologies. While this idea is interesting, it seems to me unlikely to work, because the result of this paper is based on the ability of interneurons to separately decode from spike / burst thanks to depression / facilitation. To extend this mechanism to multiple interneuron types, it seems to me that one would also need additional multiplexing in the pyramidal cell activity and additional synaptic mechanisms.
- The authors show that the specialization of inhibitory neurons in two clusters is not robust to variations of their baseline firing rate. Even though I am not an electrophysiology expert, I believe that interneurons typically have higher firing rate than excitatory ones in cortex. If the mechanism presented by the authors would work only with unrealistically low interneurons firing rate, it would significantly decrease the relevance of the work. It is also unclear if and how low firing rate of interneurons are artificial enforced during trainig (Eq. 14).

Remaining minor questions:

- Prior to training, the E-I correlation seems to be already pretty high in the soma, in contrast to the dendrites. Could the author explain the reason for this phenomenon?
- In the mathematical analysis of the simplified model, e and b are assumed to be independent. I understand that this is done for mathematical tractability, but isn't this interaction at the core of burst generation in the first place?

The manuscript is very well written and the methods are explained clearly. I only have a few minor comments regarding the text:

- There are some important details that should be included in the main text, such as the definition (at least an intuitive one) of PPR and a description of the shape of the input. Without these it is quite hard to understand the figures.
- line 111, there is a closed square bracket that should not be there.

- In the section ”Connectivity among interneurons”, I think that the authors should explain a bit more that the knockout experiments are done by removing connection types already during learning and not only after, becuase otherwise the result would be much less interesing.
- At page 8, last paragraph (after line 183), the authors write ”because those two inputs drive event rate and burst rate, respectively”. I think event and burst should be flipped in this sentence.
- In Eq. 13, the subscript i is missing from the excitatory currents.
- Line 452, independent → independently
