# Supplementary material for: Optimizing interneuron circuits for compartment-specific feedback inhibition
Source: PLoS Comput Biol. 2022 Apr 28;18(4):e1009933. doi: 10.1371/journal.pcbi.1009933 (PMC9049365; doi:10.1371/journal.pcbi.1009933)
Supplement: Attachment — Submitted filename: Compartment_specific_inhibition_PLoS_CB_reply.pdf [file pcbi.1009933.s009.pdf]

We would like to thank the reviewers for their insightful comments and constructive suggestions. Please see below for detailed responses to your concerns. We use the follow color code: *italic: reviewers' comments*; non-italic: our responses; green: changes to the manuscript. All changes to the manuscript are highlighted in the "track-changes" version of the manuscript.

Reviewer 1

The manuscript introduces an interesting approach to study the relationship between connectivity and function in spiking neural network circuits using optimization techniques. This model is used to study how specific connectivity can emerge from functional optimization required by interneurons to decode multiplexed information produced by pyramidal cells and understand the implications of different connectivity patterns in the network.

General questions

1. Were other fitness rules tested or explored? Could an XOR of the difference in excitation and inhibition between both compartments enhance the speed and performance of the optimization?

Thank you for your question. Of course it would be very interesting to study alternative objective functions, specifically objectives that are closer to functionally relevant computations than a mere E/I balance. However, this is beyond the scope of the present study. We used E/I balance as an objective, because i) it is ubiquitous in the nervous system, ii) it is directly linked to circuit stability and iii) because we have previously been quite successful in explaining circuit details and neural responses just based on E/I balance (Weber & Sprekeler, 2018; Hertaeg & Sprekeler, 2020; Mackwood et al., 2021). We tried to open up this issue at the end of the discussion (line 332):

It will be exciting to see how biological mechanisms on this level of detail support more advanced computations than the mere stabilization of the circuit considered here, but that is clearly a larger research program that extends well beyond the proof of concept presented here.

2. Which other relevant biophysical components would be interesting to add to this fitness rule in order to enhance the performance of the optimization? Which are the limitations of this rule?

Thank you for this suggestion. The optimization approach per se has little technical limitations since it relies on automatic differentiation: Any network or neuron parameter can be subject to optimization, provided it influences the objective in a smooth way. If doing so leads to interesting results is a different matter. For example, we performed simulations in which we also optimized the

time constants of the short-term plasticity and of the interneuron membranes, but neither further improved the E/I balance, nor did the optimized parameter values systematically differ from their initial values. We now added this information to the manuscript (l. 299):

Aside from the obvious incompleteness in terms of other interneuron types, other features, such as the the often observed weak connectivity from PV to SST neurons (Pfeffer et al. 2013) did not result from the optimization (Fig. 5). Our approach also did not explain further cell type-specific electrophysiological properties: Exploratory simulations indicated that optimizing membrane time constants and spike-rate adaption parameters does not further improve the compartment-specific E/I balance.

3. Why choose gradient descent? Given that the underlying dynamics are highly non linear, would this affect the shape and smoothness of the parameter space chosen, increasing the difficulty of finding unique minima? Also, the authors slightly hint that this optimization mechanism could be of evolutionary nature.

We chose gradient descent since gradient-based optimization typically outperforms gradient-free optimization. At least for high-dimensional supervised learning problems (see, e.g., Zhang, Clune, & Stanley, arXiv 2017), and as long as the gradients are well-behaved (Metz, Freeman, Schoenholz, & Kachman, arXiv 2021). Our own results in this paper and those from other groups indeed suggest that surrogate gradients of spiking networks are well-behaved and can be used for training large spiking networks. At the start of the project, we did try to minimize the objective using Bayesian optimization (Snoek, Larochelle & Adams, NeurIPS 2012), but failed to make this work. We added the following motivation for using gradient-based optimization to the manuscript (l. 542):

We used (surrogate) gradient descent instead of gradient-free methods because of its favourable sample efficiency, and its recent success in optimizing large-scale spiking networks (Bellec et al. 2018, Zenke & Vogels 2021).

We did not intend to suggest that the optimization mechanism could be of evolutionary nature, and have therefore replaced our speculations in this direction with a more cautious statement (line 278):

These developmental programs are likely old on evolutionary time scales given that interneuron classes seems more conserved than pyramidal cell classes (Tosches et al. 2018, Colquitt et al 2021). We therefore do not expect our mathematical optimization to mimic the evolutionary or developmental processes that generated interneuron diversity.

4. Which limitations are added to the optimization process by the choice that all output synapses from a given neuron to a given compartment type had the

same strength?

Thank you for raising this important question, also raised by reviewer 2. We investigated this question by optimizing networks in which interneurons connected to each pyramidal compartment with a unique strength. We found that the optimized networks still contained PV and SST clusters, but they also contained two clusters of interneurons that did not specialize for inhibiting a particular compartment. We show this result in supplementary figure 4 (see figure below), and discuss it in the renamed subsection on "Conditions for the emergence of discrete interneuron classes" (l. 180):

Finally, interneuron specialization was reduced in networks with heterogeneous inhibitory connectivity. So far, we parametrized the IN→PC connectivity such that each IN inhibited all PC somata with the same strength, and all PC dendrites with the same strength. In simulations in which interneurons were free to inhibit each soma (and each dendrite) with a unique strength, PV and SST clusters also emerged, but we additionally observed non-specialized interneurons (Fig. A.4).But do these non-specialized interneurons play an active role in the computation or are they simply not necessary and therefore left behind by the gradient descent once the problem is solved? The fact that the E/I correlation is not higher than for the homogeneous setting suggests the latter (heterogeneous networks: 0.844 +/- 0.011 (soma) and 0.702 +/- 0.022 (dendrite); homogeneous networks: 0.842 +/- 0.006 (soma) and 0.717 +/- 0.014 (dendrite)).

5. Which are the foreseen effects in the optimization process derived by the clipping of gradient values before update as well as the clipping of release values after update? Even if not in the scope of this work, it would be interesting to see which parameters does the optimization algorithm find which are not biologically realistic and which meaning they would have.

We used gradient clipping, because it's common practise and reduces the impact of occasional outliers. However, we checked now and networks trained without gradient clipping did not show different performance (compartment-specific E/I balance) or structure (PV-SST diversity).

We also trained networks without clipping the release values. This resulted in a small number of interneurons receiving net negative release probability, all of which projected strongly to the dendrites. We show the results of these simulations below, but did not include them in the manuscript, because a synapse with a negative release probability would be an inhibitory synapse that facilitates into an excitatory one, which is biologically questionable for output synapses of PCs. We did not observe initial release probabilities bigger than one in the the un-clipped experiments. Line 556

We trained networks without clipping the gradient or the release probability to confirm that this did not bias the solutions found by the optimization.

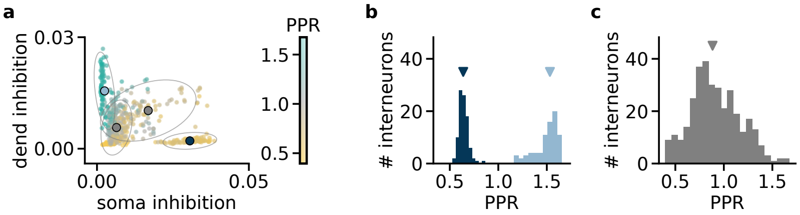

Figure 1: **Networks with heterogeneous IN→PC connections contain PV and SST classes, but also unspecific interneurons.** **(a)** IN→PC weights after optimization, in networks where INs can connect to each PC soma (and dendrite) with a unique strength. Shown are the per-IN weights averaged over all PCs. A Gaussian mixture model identified 4 clusters: a PV and a SST cluster and 2 unspecific clusters. Dots show means, ellipses show 95% density. The PV and SST clusters contain 19% and 15% of the interneurons, respectively. The unspecific clusters with small and large weights contain 52% and 14% of interneurons, respectively. **(b)** PV and SST interneurons receive depressing and facilitating inputs, respectively, as measured by the average paired pulse ratio (PPR), computed over all presynaptic PCs. Arrows indicate means. **(c)** As (b), but for interneurons of the two unspecific clusters. Unspecific interneurons do not receive a particular type of short-term plastic input.

6. Given that the optimization algorithm benefits from the network processing of input in order to better identify the generated patterns by the pyramidal cells, how do you foresee the size of the network to affect the speed and efficiency of the optimization?

We investigated this by training networks of varying sizes, comparing their optimization speed and final performance. We found that both training time and the memory storage increases approximately quadratically with network size (see Figure below). Increasing the network size while limiting the computational complexity will therefore require an algorithmic solution. We comment on this in the Methods (546):

Backpropagation through time requires storing intermediate activation values during the forward pass (network simulation), followed by a backward pass. Our network consist not just of multi-compartmental neurons, but also of a short-term plasticity model that introduces N^2 additional variables (N being the network size). Scaling the model to larger network sizes might require approximating gradient descent with a local learning rule, or gradient-free optimization.

Alternatively, using other software might decrease training time and memory storage: pilot simulations with Jax (Bradbury et al. 2018) were much faster and required less memory.

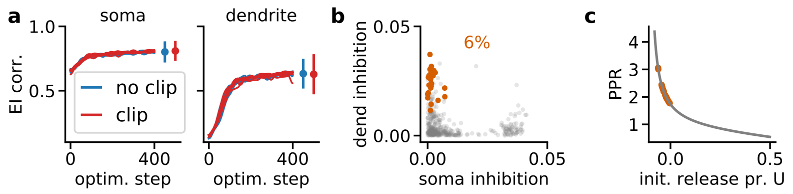

Figure 2: **Minor effect of constraining the learned initial release probability to physiologically meaningful values. (a)** Speed of learning is similar for networks trained while clipping the initial release probability to the physiological range $[0, 1]$, and for networks for which this was not done. Shown are 5 networks for each condition that differ in random initialization of their parameters. Error bars indicate mean and std over the last 10 learning trials. **(b)** Across networks, 6% of the active interneurons (25 out of n=419) develop negative initial release probabilities. All of these interneurons provide strong dendritic, but weak somatic, inhibition and would be classified as SST neurons. None of the interneurons developed an initial release probability above 1. **(c)** The few interneurons with negative initial release probabilities still receive short-term facilitating excitatory inputs, shown by the positive paired pulse ratio (PPR).

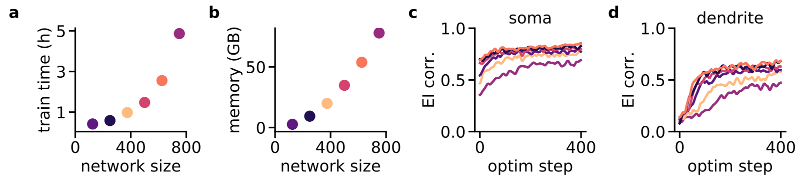

Figure 3: **Computational complexity of network optimization. (a, b)** Training time (in hours) and memory usage (in gigabytes) as a function of network size. Both were computed based on 400 optimization steps and a batch size of 8 trials. **(c,d)** Largest network has not converged after 400 optimization steps. Colors indicate network size, as shown in **(a,b)**. The results in the manuscript were obtained with a network of 500 neurons (400 PCs, 100 INs).

7. The resulting networks depend on several factors mentioned by the authors in the discussion, including plasticity rules and fitness rule chosen. Which framework would the authors suggest to put in place in order to complete a cycle with experimental data which allows to identify if the optimization results are actually correct or not?

We agree that testing the model would be an important step, and therefore expanded our paragraph with experimental predictions (l. 283):

Given these considerations, we refrain from predictions regarding the optimization process. Still, the model can make predictions regarding the nature of the optimized state. First, it predicts that excitation and inhibition are balanced on short-time scales in both somatic and dendritic compartments. Second, it predicts that PV and SST rates correlate primarily with somatic and dendritic activity, respectively. In our model, this correlation is a consequence of the decoding that underlies the balance (Fig. 2), and it is experimentally more accessible than the underlying excitatory and inhibitory currents. Third, inhibiting SST neurons should increase PC bursting, as observed in hippocampus (Royer et al. 2012) and cortex (Gentet et al. 2012). The role of short-term facilitation could be tested by silencing the necessary gene Elfn1 (Sylwestrak & Gosh 2012, Stachniak et al. 2019). On a higher level, the model suggests a relation between the biophysical properties of excitatory neurons and the surrounding interneuron circuit. This is consistent, e.g., with the finding that the prevalence of pyramidal cells and dendrite-targeting Martinotti cells seems to be correlated across brain regions (Scala et al. 2019). This and other correlations between interneuron and pyramidal properties could be investigated systematically using recent electrophysiological and genetic data from different areas and organisms (Gouwens et al. 2020, Berg et al. 2021, Beaulieu-Laroche et al. 2021).

Devising a framework for closing the theory-experiment loop seems a laudable but also ambitious goal. We therefore think it is outside the scope of the paper.

8. How would the fitness rule change if the number of compartments change? Do you still think the current approach would hold?

Thank you for raising this point regarding the general applicability of our approach. Reviewer 2 raised a similar point. As they rightfully pointed out, we rely on the multiplexing and demultiplexing of soma/dendrite specific information, but is is presently unclear how this mechanism would generalize to multiple compartments. Explaining a greater scope of interneuron diversity therefore likely requires a fitness function other than a compartment-specific E/I balance. We think it is important that readers are aware of this limitation, and have therefore emphasized it now in the discussion (l. 308):

It will be interesting to see how the suggested optimization framework generalizes to computations performed by more complex neuronal morphologies (Poirazi et al. 2020). A key challenge in this regard will be the choice of an appropriate computational objective. The objective of E/I balance across different compartments may not generalize to more complex morphologies, because it is unclear if pyramidal cells can multiplex across more than two compartments – because that would require more different spike patterns – let alone that interneuron circuits could invert such a neural code.

9. The authors mention in the discussion that this optimization approach

would be useful to analyze the structural and functional relationships of other spiking neural circuits and also could be extended to cells with more compartments. In order to enable this and for reproducibility of the manuscript, the authors should share their code and data used for the analysis and plots though a public repository. Specific versions of the software used e.g. Tensorflow and Scikit-learn are also missing.

Thank you, we forgot the software versions but added them now (line 526). We agree that the availability of data and code is important, and therefore had already provided both in the original manuscript (Under "Code Availability"). We will upload the data and code to the public repository https://github.com/sprekelerlab/ upon publication.

Small typos

Equation 13 is missing the subscript i in E_i^s *and* E_i^d*.*
In the methods for figures section, PPR and EPSP are used before they are defined.
L452 ...optimized independently...

Thank you, we have now fixed these oversights.

Reviewer 2

The authors proposed a normative explanation for the diversity of inhibitory interneurons in the cortex. More precisely, they hypothesize that different interneurons types emerge from the objective of balancing excitation and inhibition at a compartmental level. The authors test the feasibility of their hypothesis using an optimization approach, and show that in some cases two clusters (one per model neuron compartment) emerge from an initially homogeneous group of neurons. The main result is illustrated in Fig. 1 and most of the paper is dedicated to understanding the underlying mechanisms and to the robustness of this phenomenon. One of the most interesting observations in my opinion is that to achieve the objective, interneurons should be able to "invert" the spiking signal to obtain the membrane potential. This problem is elegantly solved in the model by specializing different interneurons to read out only bursts or only spikes thanks to short-term plasticity. However, it should be pointed out that this idea is not entirely new, as the authors themselves reference previous work from the same group (Naud and Sprekeler, 2018), in which the same basic mechanism was already described. The main new contribution of this paper is then the application of this mechanism to the compartment specific balance of excitation and inhibition. *I see a few major problems with the conclusions that the authors are drawing:*

Are there any other solutions of the compartment specific E/I balance that

do not involve short-term plasticity? This could be easily tested by removing STP from the optimization.

Thank you for raising this important point. In our model, STP is indeed necessary for the compartment-specific balance. We knew this from hand-wired circuits (Naud & Sprekeler 2018, their supplementary figures 8 and 9), and had tested this by training networks without STP, but obviously failed to highlight this in the manuscript. We only mentioned the need for STP briefly in the context of Fig. 4d and Suppl. Fig. 2. STP is not as crucial for a somatic balance, but it is for a dendritic balance (see also reviewer's first minor comment, below). We have now expanded on this aspect (line 149, Figures 3f and A.3, shown after text excerpt):

In networks trained without short-term plasticity, SST neurons were not selectively activated by bursts, and therefore dendritic inhibition did not balance dendritic excitation (Figs. 3f, A.3). A soma-specific E/I balance also required short-term plasticity, but only for weak somatic excitation—consistent with the relatively high somatic E/I correlation at the start of training (Fig. 1). In the networks trained without short-term plasticity, the multiplexed neural code was unaltered, because the biophysics of the PCs are the same, but the decoding by the interneuron circuit fails, most prominently for the dendritic input. In our model, short-term plasticity is therefore necessary for a compartment-specific feedback inhibition.

Would two clusters of interneuron emerge in a heterogeneous network in which the inhibitory connectivity is allowed to take any value (and not the same for all neurons)?

This important question was also brought up by reviewer 1. We addressed it by training heterogeneous networks in which the interneuron connected to each pyramidal compartment with a unique strength. We found that these networks do contain PV and SST clusters, but also interneurons that do not obviously fall into either category (supplementary figure 4, see figure below). We mention this result in the section now titled "Conditions for the emergence of discrete interneuron classes" (line 180):

Finally, interneuron specialization was reduced in networks with heterogeneous inhibitory connectivity. So far, we parametrized the IN→PC connectivity such that each IN inhibited all PC somata with the same strength, and all PC dendrites with the same strength. In simulations in which interneurons were free to inhibit each soma (and each dendrite) with a unique strength, PV and SST clusters also emerged, but we additionally observed non-specialized interneurons (Fig. A.4). But do these non-specialized interneurons play an active role in the computation or are they simply not necessary and therefore left behind by the gradient descent once the problem is solved? The fact that the E/I correlation is not higher than for the homogeneous setting suggests the latter (heterogeneous

networks: 0.844 +/- 0.011 (soma) and 0.702 +/- 0.022 (dendrite); homogeneous networks: 0.842 +/- 0.006 (soma) and 0.717 +/- 0.014 (dendrite)).

In the discussion, the authors ask whether it would be possible to extend their results to multiple interneuron classes by exploiting more complex neuronal morphologies. While this idea is interesting,it seems to me unlikely to work, because the result of this paper is based on the ability of interneurons to separately decode from spike / burst thanks to depression / facilitation. To extend this mechanism to multiple interneuron types, it seems to me that one would also need additional multiplexing in the pyramidal cell activity and additional synaptic mechanisms.

We agree that a straightforward generalization of our approach based on multiplexing is unlikely to work. We also did not intend to suggest this, but rather that the general approach of optimizing synaptic, cellular or circuit properties for a given function might be broadly applicable. We clarified this in the revised manuscript (line 308):

It will be interesting to see how the suggested optimization framework generalizes to computations performed by more complex neuronal morphologies (Poirazi et al. 2020). A key challenge in this regard will be the choice of an appropriate computational objective. The objective of E/I balance across different compartments may not generalize to more complex morphologies, because it is unclear if pyramidal cells can multiplex across more than two compartments – because that would require more different spike patterns – let alone that interneuron circuits could invert such a neural code.

The authors show that the specialization of inhibitory neurons in two clusters is not robust to variations of their baseline firing rate. Even though I am not an electrophysiology expert, I believe that interneurons typically have higher firing rate than excitatory ones in cortex. If the mechanism presented by the authors would work only with unrealistically low interneurons firing rate, it would significantly decrease the relevance of the work.

The reviewer is correct that (PV) interneurons typically have higher firing rates than excitatory ones, but based on the mathematical analysis, we believe that it is not the baseline firing rate per se that matters, but rather the relation of the baseline activity and the dynamic range required to compensate "erroneous" inhibition that arises in a non-specialized setting. This is a subtle point that we now try to clarify in the main text as follows (line 171):

Interneuron specialization also degraded with increasing baseline activity of the INs (SI Appendix, Fig. A.2), because high firing rates allow non-specialized inhibition to be canceled by equal and opposite disinhibition (see mathematical analysis in Section (SI Appendix, Computational Methods). The dependence on baseline activity results from the fact that disinhibition is limited by how much the firing of the interneurons can be reduced. In that regard, it is not

the baseline firing rate itself that determines the specialization – which is often higher for interneurons than for PCs (see, e.g., Yu et al. 2019) –, but the relation between the baseline and the dynamic range of the firing rates that is required for the appropriate disinhibition. Note also that a pre-assignment of interneurons into classes again maintained the E/I correlation for different baseline activity levels (SI Appendix, Fig. A.1).

In that light, we believe that this constraint does not really decrease the relevance of the work, because interneurons may often have higher baseline firing rates, but also a larger dynamic range (e.g., "fast-spiking" PV interneurons with their maximum firing rate).

It is also unclear if and how low firing rate of interneurons are artificial enforced during trainig (Eq. 14).

Yes, the minimum firing rate is enforced indirectly, by varying the "baseline" target in the objective function for somatic inhibition in the absence of somatic excitation. Mathematically, the target for the inhibitory currents is the excitatory current minus the minimum excitatory current (equation 14). After optimization, the interneurons will therefore rarely fire during moments when the excitatory current is at its baseline. We extended the explanation of this procedure in the Methods (Line 544):

To achieve small interneuron rates necessary for interneuron specialization (Fig. A.2), we subtracted the mean background input (...) such that interneurons did not fire when their target compartment received its minimum level of external excitation.

Enforcing higher interneuron baseline rates is done by subtracting only a fraction of the minimum excitatory current (Equation 18, line 611). This approach has the advantage that it allows an – albeit indirect – control of the interneuron firing rates after convergence.

minor comments

Prior to training, the E/I correlation seems to be already pretty high in the soma, in contrast to the dendrites. Could the author explain the reason for this phenomenon?

The reason is that event rate and firing rate are highly correlated (Naud & Sprekeler, 2018), while burst fraction and firing rate are not. Because those terms have unfortunately not been introduced at the most appropriate point in the manuscript, we only added the following to the results section (line 105):

The relatively high correlation between somatic excitation and inhibition is explained by the fact that, in a recurrent network, inhibition is bound to track

excitation to some extent (Van Vreeswijk et al. 1996, Renart et al. 2010).

In the mathematical analysis of the simplified model, e and b are assumed to be independent. I understand that this is done for mathematical tractability, but isn't this interaction at the core of burst generation in the first place?

You are correct, the interaction is at the core of burst generation, making it an aspect of the simulations not captured by the simplified model. Unfortunately, this interaction is nonlinear and cannot be treated analytically. The math could therefore only be considered as a linearization of the true nonlinear system. We now briefly comment on this point in the main text (l. 207):

The activity of PCs is described by two rates: an event rate e that is driven by somatic input and a burst rate b that is driven by dendritic input. The burst rate is assumed to be independent of the somatic input, which is different from BAC firing (Larkum et al. 1999), but generates a linear model that is analytically tractable.

That being said, the math did provide us with insights into the conditions under which a specialization is expected, which match the simulations quite well. In that sense, the math may be a simplification, but it was quite useful for us. As an aside, we also implemented a simpler version of the network that is closer to the theory (we'll spare you the details) that also generates two interneuron classes.

There are some important details that should be included in the main text, such as the definition (at least an intuitive one) of PPR and a description of the shape of the input. Without these it is quite hard to understand the figures.

Thank you for letting us know that certain details were lacking. We now added a brief explanation of the PPR and shape of the input (lines 86 and 100):

PCs received time-varying excitatory inputs in both the somatic and the dendritic compartment, and inhibitory inputs from INs. The excitatory inputs to both compartments consisted of alternating currents of varying amplitude.

Synaptic dynamics were quantified using the paired pulse ratio (PPR), the relative amplitude of consecutive postsynaptic potentials (see Methods). A PPR smaller than 1 indicates that later postsynaptic potentials are weaker, and therefore corresponds to short-term depression.

In the section "Connectivity among interneurons", I think that the authors should explain a bit more that the knockout experiments are done by removing connection types already during learning and not only after, becuase otherwise the result would be much less interesing.

Thank you for this suggestion, we added the following explanation (line 198):

To investigate which connections were necessary, we simulated knockout experiments in networks with pre-assigned interneuron classes, in which we removed individual connections types prior to optimization

At page 8, last paragraph (after line 183), the authors write "because those two inputs drive event rate and burst rate, respectively". I think event and burst should be flipped in this sentence.

You are correct, it should be flipped. We did so in the revised manuscript (line 216).

In Eq. 13, the subscript i is missing from the excitatory currents. Line 452, independent -independently

Thank you, we have now made these corrections.

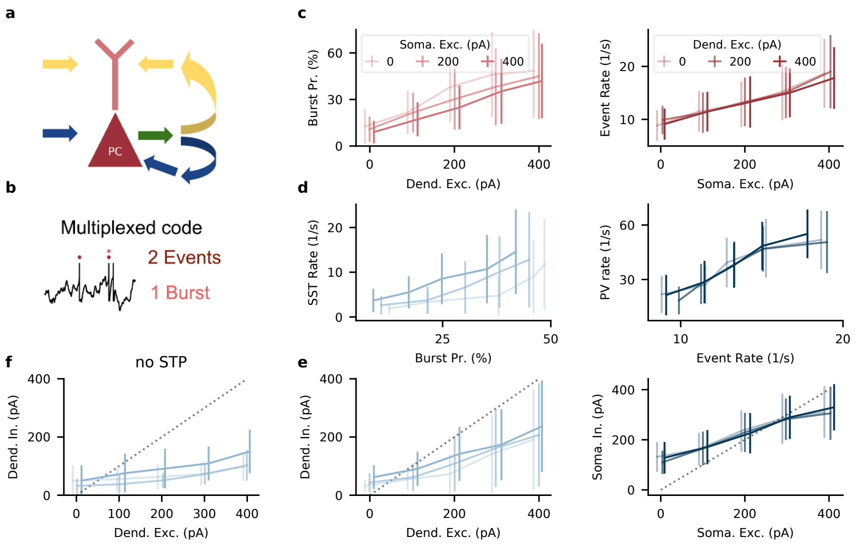

Figure 4: **The interneuron circuit decodes somatic and dendritic inputs to PCs.** **(a)** PC somata and dendrites receive uncorrelated input streams (yellow and blue) that, from PC output spikes (green), have to be separated into compartment-specific inhibition (yellow and blue). **(b)** PCs use a multiplexed neural code. Somatic input leads to events (singlets or bursts). Dendritic input converts singlets into bursts. **(c)** Left: Excitatory input to PC dendrites increases burst probability. In this and other top panels (d,e), the shading indicates strength of background somatic input. Right: Excitatory input to PC somata increases event rate. In this and other bottom panels (d,e), the shading indicates strength of background dendritic input. **(d)** Left: SST rate increases with bursts probability. Right: PV rate increases with PC events. **(e)** Left: dendritic inhibition increases with dendritic excitation, but is only weakly modulated by somatic excitation. Positions on x-axis are shifted by 10 pA for visual clarity, error bars indicate sd during 10 stimulus repetitions. Right: somatic inhibition increases with somatic excitation, but is invariant to dendritic excitation. Dashed lines correspond to excitation = inhibition. **(f)** In networks trained without short-term plasticity, dendritic inhibition shows a weaker dependence on dendritic excitation and a stronger dependence on somatic excitation. Also see supp. Fig. A.3

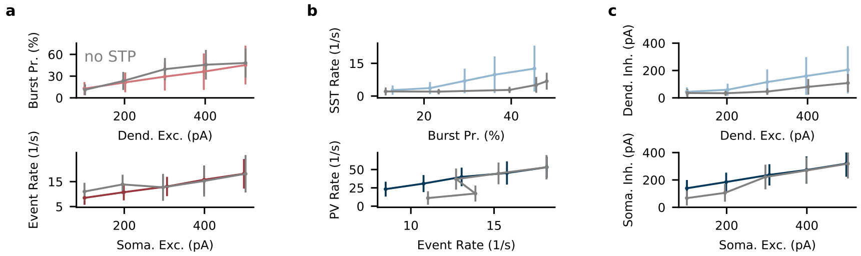

Figure 5: **Networks without short-term plasticity fail to achieve a dendrite-specific E/I balance. (a)** PCs use a multiplexed neural code both in presence (colors) and absence (gray) of short-term plasticity in their efferents. Top: Excitatory input to PC dendrites increases burst probability. Bottom: Excitatory input to PC somata increases event rate. **(b)** Top: SST rate increases with bursts probability only when SSTs receive short-term plastic input. Bottom: PV rate increases with PC events, but absent short-term plasticity only for intermediate and high event rates. **(c)** Top: dendrite-specific inhibition requires short-term plasticity. Bottom: soma-specific inhibition requires short-term plasticity only for small somatic input.

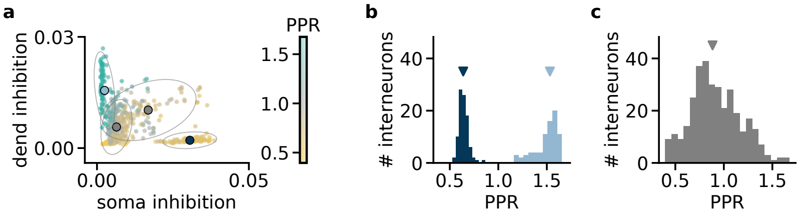

Figure 6: **Networks with heterogeneous IN→PC connections contain PV and SST classes, but also unspecific interneurons. (a)** IN→PC weights after optimization, in networks where INs can connect to each PC soma (and dendrite) with a unique strength. Shown are the per-IN weights averaged over all PCs. A Gaussian mixture model identified 4 clusters: a PV and a SST cluster and 2 unspecific clusters. Dots show means, ellipses show 95% density. The PV and SST clusters contain 19% and 15% of the interneurons, respectively. The unspecific clusters with small and large weights contain 52% and 14% of interneurons, respectively. **(b)** PV and SST interneurons receive depressing and facilitating inputs, respectively, as measured by the average paired pulse ratio (PPR), computed over all presynaptic PCs. Arrows indicate means. **(c)** As (b), but for interneurons of the two unspecific clusters. Unspecific interneurons do not receive a particular type of short-term plastic input.
